# Generation of a 28 GHz annular power distribution with high power gyrotron and its application to microwave driven in tube accelerator

**DOI:** 10.1038/s41598-025-08430-3

**Published:** 2025-07-07

**Authors:** Masayuki Takahashi, Toshiki Yamada, Ryutaro Minami, Tsuyoshi Kariya, Kohei Shimamura

**Affiliations:** 1 Department of Aerospace Engineering, Tohoku University, Sendai, 980-8579 Japan; 2 Plasma Research Center, University of Tsukuba, Tsukuba, 305-8577 Japan; 3 Department of Aeronautics and Astronautics, Tokyo Metropolitan University, Hino, 191-0065 Japan

**Keywords:** Tractor millimeter-wave beam, Plasma, Beamed energy propulsion, Aerospace engineering, Electrical and electronic engineering

## Abstract

The microwave-driven in-tube accelerator (MITA) concept, which installs the center body of the thruster inside the waveguide and generates thrust via millimeter-wave beam irradiation from the front side of the center body, was experimentally demonstrated using 210-kW and 28-GHz gyrotron device. For the 28-GHz beams, a vortex phase plate was newly designed to change the incident beam profile from a Gaussian to a donut-shaped pattern, which was installed in front of the thruster’s center body. Without the vortex phase plate, gas breakdown occurred at the center-body head, providing a negative impulse. However, as the electric field concentration at the center-body head was avoided using the vortex phase plate, plasma and strong shock wave generation were obtained at the rear side of the vehicle, inducing a positive impulse to accelerate the thruster toward the beam source direction. The MITA thrust performance increased as the incident beam pulse width increased because gas heating was enhanced at the rear side of the center body.

## Introduction

Microwave rocket technology, which obtains a propulsive force by receiving an intense millimeter-wave pulse irradiated from a ground oscillator to the vehicle, has been proposed to reduce space transportation costs^1–31^. The incident electromagnetic beam is focused inside the thruster nozzle, inducing gas breakdown and shock waves that provide thrust to the rocket. On-board fuel can be reduced via propulsive energy transmission from an external gyrotron device, reducing transportation costs^32–36^. The engine structure is simplified, and a complex gas-burning engine system is not needed for thrust, increasing the system’s reusability and decreasing launch cost. A gyrotron device is a vacuum tube converting electron kinetic energy along magnetic field lines into millimeter-wave energy. It achieves high output power of the MW class among millimeter-wave oscillators. In the gyrotron device, high voltage is applied to the electron gun to draw out high-energy electron beam. This electron beam enters the cavity resonator in spiral motion along magnetic fields created by a superconducting magnet. The rotational energy of electrons converts to millimeter-wave energy in the resonator, radiating out through an optical window for use as microwave rocket power source.

A previous demonstration of a microwave rocket indicated that dense plasma and shock waves were induced inside the rocket nozzle via a gas breakdown process when a millimeter-wave beam was irradiated onto the vehicle^1–4,13^. The detailed plasma and shock wave structures inside the nozzle were captured using a high-speed camera, revealing that fascinating plasma structures such as fish-bone, diffusive, and branching patterns were induced during the millimeter-wave discharge process^7–12^. Furthermore, the microwave rocket thrust performance was measured using an impulse measurement device^1–4,13^. Although a relatively high thrust performance, which is effective for rocket launching, was obtained for single-pulse irradiation, its performance decreased with time under repetitive pulse operation because the previously generated plasma did not disappear from inside the nozzle and screened the next beam irradiation. Consequently, the plasma ignition position moved toward the beam source direction via repeated pulse irradiation, which eventually protruded from the nozzle and did not contribute to thrust generation. Besides the decrease in axial thrust, although flight control technology has been proposed, as indicated in previous works^32–35^, maintaining flight stability is difficult during free flight, hindering the establishment of this launch system. Therefore, based on an analogy with the laser-driven in-tube accelerator (LITA)^36^, the microwave-driven in-tube accelerator (MITA) concept was proposed to avoid the thrust performance degradation during repetitive pulse operation and difficulties in flight control^21,27^.

**Figure 1:**
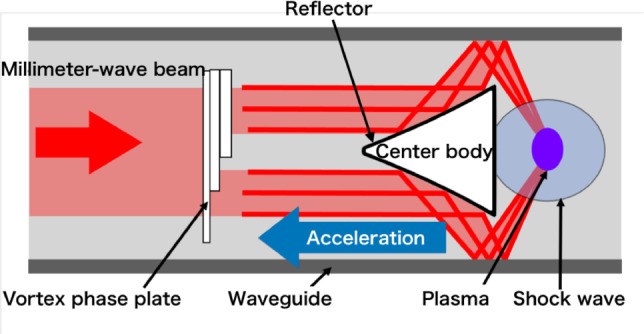
Schematic of the MITA with a vortex phase plate.

In the MITA concept, a cone-shaped center body with a beam reflector is installed inside the waveguide to limit the flight motion degree-of-freedom (DOF) to one dimension, as shown in Fig. 1; therefore, flight instability can be avoided by accelerating the center body inside the waveguide. The payload mass is installed inside the center body. Repetitive millimeter-wave pulses are irradiated from the front to the rear sides of the vehicle, which are reflected on the front surface of the center body, and their propagation direction is changed. Subsequently, the electromagnetic wave beam is reflected on the waveguide surface while achieving beam focusing on the rear side of the center body. A high-electric-field region is obtained at the rear side of the center body, which could induce gas breakdown and shock waves. The shock wave high pressure interacts with the rear surface of the center body, generating thrust in the beam source direction. Because beam transmission is conducted in the waveguide, significant beam divergence during transmission can be avoided. The corrugated waveguide, a circular waveguide with circumferential grooves in the inner wall, is most promising for transmission. In corrugated waveguides, transmission occurs in the low-loss $\hbox {HE}_{11}$ mode, but this study did not reproduce this mode in the thruster tube; therefore, waveguide design should be considered for actual MITA operation. According to conventional corrugated waveguide technology, instead of beam divergence in the transmission line, approximately 40% energy loss can occur with a 400-m transmission line^37^. However, gas breakdown and shock wave generation are possible when a beam source of more than 200 kW is used. After vehicle acceleration in the 400-m beam transmission line, the flight mode must be switched from in-tube acceleration to free flight; therefore, the MITA system can be used as an initial mass driver for rocket launching. Moreover, as another use of the MITA system, a space elevator driver can be considered.

For the MITA concept, we previously conducted a coupling simulation using finite-difference time-domain (FDTD), plasma reaction transport, and computational fluid dynamics (CFD) modules to confirm the feasibility of the thruster^21,27^. The simulation results revealed that the electromagnetic wave beam irradiated from the front side of the center body was successfully focused on the rear side of the center body, inducing gas breakdown and dense plasma formation. A high-electron-temperature region is generated owing to Joule heating. The shock wave was induced via energy transmission from the plasma to neutral particles, which interacted with the rear surface of the center body and generated thrust. However, a MITA experimental demonstration has not been conducted using a high-power millimeter-wave beam source. Furthermore, its thrust performance and experimental validity were not evaluated in beam irradiation experiments.

Accordingly, in this study, a breakdown experiment for the MITA was performed using a 28-GHz gyrotron deceive with a Gaussian beam profile to assess its thrust performance. The beam was irradiated from the front of the MITA, and the plasma-generation process inside the cylindrical tube was observed using a digital camera. The generated MITA impulse was measured using a pendulum-type impulse measurement device developed in house. In the beam irradiation experiment, a vortex phase plate^38–45^ was newly developed and introduced in front of the center body to change the beam profile from Gaussian to a donut shape, avoiding unexpected gas breakdown at the top of the center body, as shown in Fig. 1. The plasma structure and thrust performance were compared between the thruster models with and without the vortex phase plate. Finally, the incident beam pulse width ($\tau$) was changed to conduct a parametric study. For plasma and shock wave generations, the working gas inside the waveguide can be selected arbitrarily. The thrust performance may increase with the molecular weight of the working gas, as discussed in previous work for the LITA^36^. The pressure of the working gas is a controllable parameter, with a tradeoff between aerodynamic drag on the center body and thrust performance. Understanding the effects of gas species and pressure on thrust performance is important, but in this study, the working gas was set as air at 1-atm pressure.

Vortex phase plates used in this study was suggested to change the beam mode^38–40^. Previous experiments using high-power gyrotron devices showed that donut-shaped beam generation with orbital angular momentum (OAM) is possible by irradiating the Gaussian beam onto the vortex phase plate^41–45^. Theoretical and experimental studies for OAM beam generation showed that vortex phase plate design can change the topological charge of beam *l*, which determines the OAM. We focused on the OAM beam with $l=1$ mode as the first step of MITA thrust generation; however, in generating the donut-shaped profile beam, there are degrees of freedom in beam mode, and other modes can be candidates for energy sources in the MITA concept. The use of other modes is discussed in the conclusion section.

## Results and discussion

### Plasma structure without a vortex phase plate

**Figure 2:**
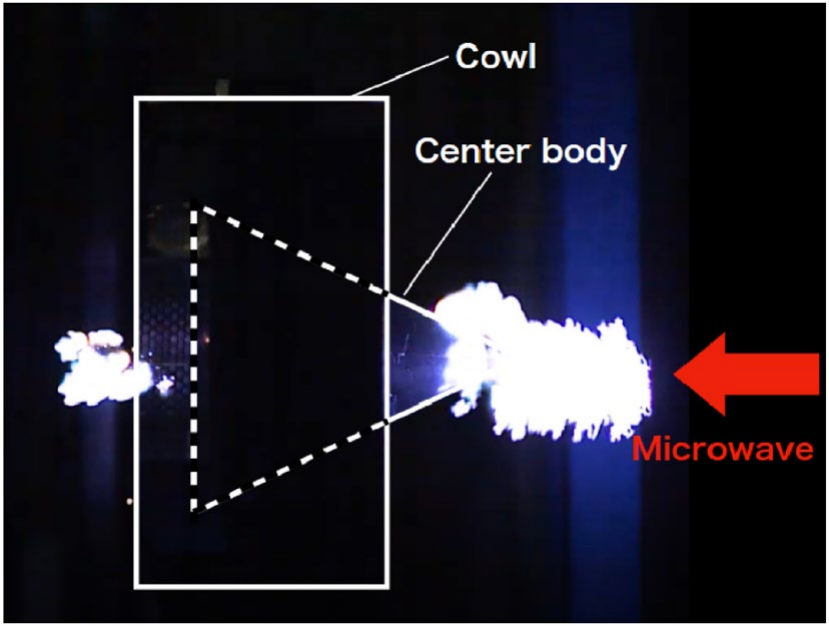
Integral image of plasma without a vortex phase plate.

Figure 2 shows an integral image of the plasma when the vortex phase plate is removed from the thruster. A beam with a Gaussian-like profile directly reaches the cone-shaped center body. Although successful gas breakdown was predicted only at the rear of the center body in previous numerical simulations^21,27^, our beam irradiation experiment revealed that gas breakdown occurred at both the rear and front sides of the center body. This is because the incident electromagnetic wave beam was concentrated at the top of the center body, inducing strong electron-impact ionization. Because the electric field enhancement at the center-body top and the sequential ionization process were not considered in the simulation, there was a difference between the simulated and experimental results. In this thruster model, because the plasma generated at the center body front absorbed the incident beam energy, strong gas heating occurred, pushing the center body from the beam source in the thruster rear direction. Consequently, the impulse measurement results indicate that the net impulse was negative. To obtain a positive net impulse, it is necessary to avoid electromagnetic wave enhancement at the top of the center body. Therefore, a vortex phase plate was designed and placed in front of the center body, creating a donut-shaped beam profile and avoiding the generation of a high electric field at the top of the center body.

### Plasma structure and impulse with a vortex phase plate

**Figure 3:**
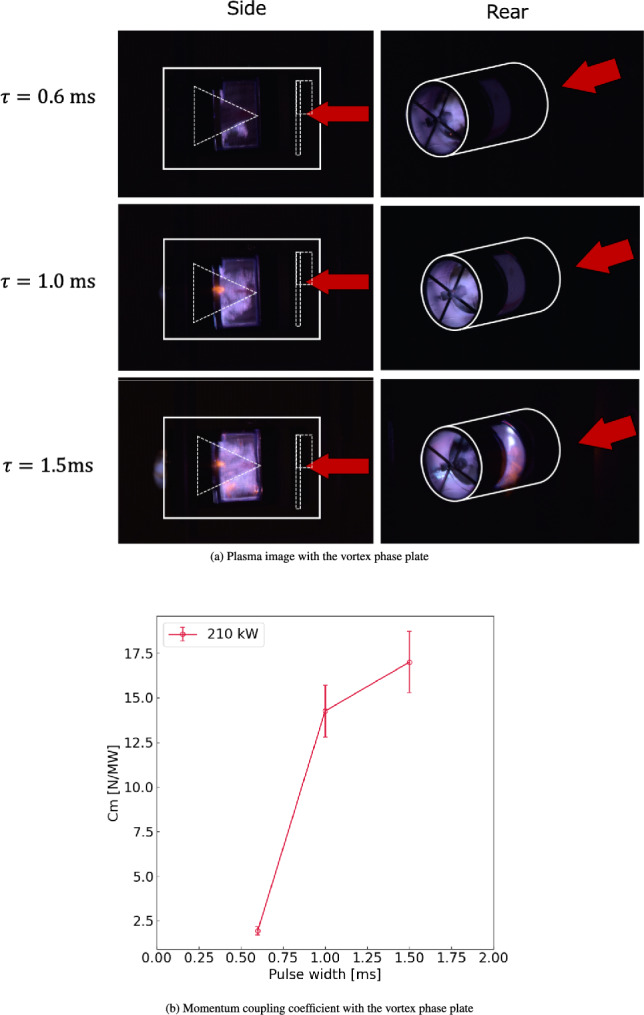
Integral images of plasma and momentum coupling coefficient with the vortex phase plate. $\tau$ is the pulse width.

Figure 3a shows an integral image of the plasma structure obtained by placing a vortex phase plate in front of the center body. As shown for a pulse width of $\tau =0.6$ ms, gas breakdown and strong light emission at the top of the center body were avoided when the vortex phase plate was mounted in front of the center body because the electric field intensity along the center body axis decreased. Besides avoiding the strong gas breakdown at the center-body top, the gas breakdown and plasma light emission at the rear side of the center body were successfully achieved, as shown in the image from the center-body rear at $\tau =0.6$ ms. This plasma generation at the center-body rear could induce gas heating and shock waves via the Joule heating process. The high pressure of the shock wave interacts with the rear surface of the center body, pushing the thruster in the beam-source direction. Therefore, as shown in Fig. 3b, the net impulse (momentum coupling coefficient) becomes positive when the vortex phase plate is mounted, confirming that our MITA concept, which generates thrust using a tractor millimeter-wave beam irradiated from the front side of the vehicle, was experimentally achieved.

Furthermore, as a parametric study, changes in the plasma generation dynamics and impulse were evaluated by increasing the pulse width $\tau$ from 0.6 to 1.5 ms. The side views for $\tau =0.6$ and 1.0 ms (Fig. 3a) show that a spatial area, where the plasma light emission is observed, was extends from the center-body rear to the front’s reflector (the oblique area of the cone-shaped center body), while avoiding strong light emission and gas breakdown at the top of the center body. This plasma front propagation toward the reflector area of the center-body front was caused by the ionization process owing to the high electric field around the front reflector. A detailed plasma front propagation process could be explained by a combination of a gas temperature increase, reduced electric field increase, heavy particle excitation, and consequent thermal and accumulative ionization, as discussed in previous simulations studies^18,29–31^. The plasma around the front reflector can induce a shock wave, pushing the center body in the negative impulse direction. However, the shock wave induced at the rear of the center body (around the focal point of the reflector) was stronger because of the electric field focus and concentration, which contributed to the generation of a larger positive impulse. Additionally, the shock wave induced around the front reflector can push the vortex phase plate in the positive impulse direction. As the vortex phase plate was connected to the center body, this positive impulse was also applied to the center body. Because the positive impulse generation on the center-body rear and the vortex phase plate surfaces were larger than the negative impulse generated on the reflector surface, $C_m$ at $\tau =1.0$ ms was larger than that at $\tau =0.6$ ms, as shown in Fig. 3b. Furthermore, as the pulse width increased ($\tau =1.5$ ms), the gas heating at the center-body rear became stronger, as indicated by the stronger light emission at the rear image of the center body ($\tau =1.5$ ms in Fig. 3a), generating the larger positive thrust. Additionally, a stronger shock wave interaction occurred with the vortex phase plate at $\tau =1.5$ ms, increasing $C_m$. Therefore, as a general trend for thrust performance, $C_m$ increased as the incident beam pulse width increased when the vortex phase plate was mounted on the thruster.

Although further performance improvements can be achieved by increasing the pulse width to more than 1.5 ms, this, along with the optimal conditions for thrust performance, will be discussed in future work. Additionally, because it is assumed that the aerodynamic drag force can be increased in the present thruster design owing to the flat shape of the vortex phase plate, an aerodynamic design for the entire transportation system should be addressed for future launch missions. Although an air-breathing methodology should be designed, these aerodynamic aspects and air-breathing designs will be discussed in future work.

## Conclusion

The MITA concept was experimentally demonstrated using 28-GHz and 210-kW gyrotron devices. A vortex phase plate was designed to prevent gas breakdown at the thruster head by changing the Gaussian-like beam profile into a donut-shaped beam profile. The thruster without the vortex phase plate generated gas breakdown and strong light emission from the plasma at the thruster head, inducing a negative impulse. However, a donut-shaped beam profile was created by mounting the vortex phase plate in front of the center body, avoiding gas breakdown at the top of the center-body front. Additionally, plasma generation at the rear side of the center body was successfully achieved using a vortex phase plate, inducing a shock wave. This shock wave interacted with the rear surface of the thruster center body, generating a positive impulse and confirming that our MITA concept was experimentally achieved. Furthermore, a parametric study was conducted by increasing the indecent beam pulse width from 0.6 to 1.5 ms. As the pulse width increased, the plasma-generation region extended toward the front side of the center body; however, the net impulse was still positive because the electric field concentration at the top of the center body was avoided. As a general trend for thrust performance, the momentum coupling coefficient increased as the pulse width increased because the gas heating at the center-body rear side became stronger, contributing to a larger positive thrust. Additionally, as the pulse width increased, the high-temperature gas interacted more strongly with the vortex phase plate surface, contributing to an increase in the momentum coupling coefficient.

In the MITA concept, a donut-shaped beam with topological charge $l=1$ was irradiated onto the center body to avoid gas breakdown at the center body top; however, in generating a donut-shaped beam, different beam modes can be used. Here we discuss using such other modes of beams with donut-shaped profiles for impulse generation. Vortex beams with topological charge $l = 1$ or $l = 2$ induced by a vortex phase plate both have a donut-shaped profile usable for the MITA concept. Although the plasma structure may change when an $l= 1$ or an $l = 2$ vortex beam generated from the $\hbox {HE}_{11}$ mode by a phase plate or a circular symmetric $\hbox {TE}_{01}$ mode beam with $l = 0$ is irradiated for impulse generation of MITA, we believe the time-averaged beam density distribution is more important than the beam’s OAM. Previous experimental and modeling studies^4,20^ indicated that the shock wave strength induced by the millimeter-wave discharge and the thrust impulse are strongly influenced by the incident beam energy and the Joule heating at the plasma front, rather than by the plasma and beam mode structure. The $\hbox {TE}_{01}$ mode can produce a different donut-shaped profile than the $l = 1$ or $l = 2$ modes generated by vortex phase plates, and may eventually produce a higher impulse. However, the important factors are the time-averaged donut-shaped beam profile and the Joule heating rate at the focal point. We will verify these speculations in future experiments using the $\hbox {TE}_{01}$ mode or a different topological charge ($l = 2$) from the corresponding vortex phase plate.

## Methods

### Thruster model and vortex phase plate

**Figure 4:**
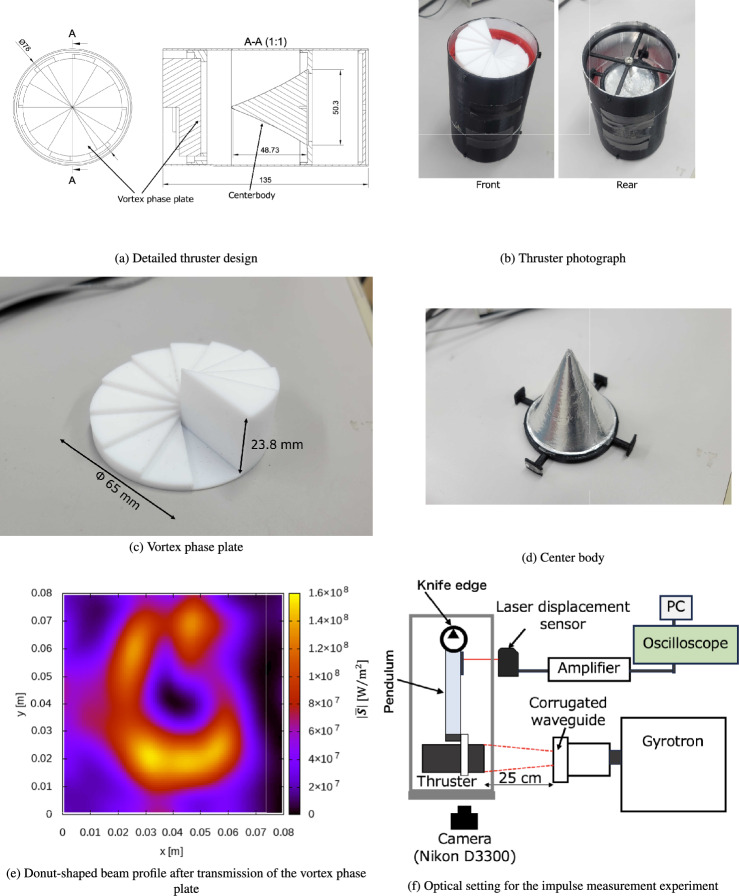
Thruster model, vortex phase plate, and optical setting.

The thruster model was designed based on a previous experiment that used the LITA concept^36^. The shape of the cone-shaped center body was the same as that proposed by the LITA system, as shown in Fig. 1, which had an incident beam reflector on its front side. Although the original LITA design was for laser beam irradiation with a wavelength shorter than the millimeter-wave beam, its validity in terms of beam-focusing performance was demonstrated for millimeter-wave beam irradiation based on previous simulations^21,27^. The cone-shaped center body with a beam reflector was installed in a cylindrical tube, and the beam reflected on the front reflector was reflected on the tube surface again. Finally, beam focusing was achieved at the rear side of the center body, which induces gas breakdown. Figure 4a,b show the detailed MITA design and its photograph, respectively. A vortex phase plate (Fig. 4c), which can change the incident beam profile from Gaussian to a donut shape, was placed in front of the cone-shaped center body. Using holders, the vortex phase plate and cone-shaped center body (Fig. 4d) were fixed to the cylindrical tube and installed inside the waveguide in the actual launch system. The ignition pin of the M4 bolt was placed around the focal point of the reflector at the center body. The vortex phase plate was removed from the cylindrical tube to compare the thrust performance. The center body, cylindrical tube, and holders were fabricated from PLA resin using a 3D printer (Finder3, FLASHFORGE). Aluminum tape was placed on the center body surfaces and the inner surface of the cylindrical tube to reflect the incident millimeter-wave beam.

The vortex phase plate has a staircase shape (Fig. 4c), which gradually changes the phase of the incident beam along the azimuthal direction of the beam axis^38–40^. It was designed so that the total phase shift became an integer multiple of 2$\pi$. Because of this phase shift, an optical vortex was generated with a donut-shaped beam profile. The thickness difference between the thickest and thinnest parts of the vortex phase plate was defined as *h*, where *h* should be an integer multiple of the incident beam wavelength $\lambda$. In the phase plate, *h* is expressed as

1 $$\begin{aligned} h=\frac{\lambda l}{\sqrt{\epsilon _r}-1}, \end{aligned}$$

where $\epsilon _r$ is the relative permittivity of the phase plate and *l* is the topological charge having an integer value^40,44,45^. In this study, the incident beam wavelength was 10.7 mm for the 28-GHz beam. Polytetrafluoroethylene (PTFE) was used for the vortex phase plate, and $\epsilon _r$ was 2.1 for the PTFE; therefore, *h* was designed as 23.8 mm for $l=1$. As the phase plate used in this study had a spiral staircase circling in 12 steps, the height of a single staircase was 2.16 mm. The vortex phase plate diameter was 65 mm.

For the vortex phase plate designed for 28-GHz beams, we previously conducted a three-dimensional (3D) FDTD simulation with Mur absorbing boundary conditions^46^, which indicated that the Gaussian beam profile can be converted to a donut-shaped beam profile after transmission of the vortex phase plate, as shown in Fig. 4e. The simulation code was based on our previous works^18,21,29–31^. In the FDTD simulation, a 28-GHz Gaussian beam with a beam waist of 20.3 mm was irradiated onto the vortex phase plate, whose shape was the same as that used in the experiment. The distance between the beam source modeled in the simulation and the vortex phase plate was set to 250 mm, which was the same as that in the breakdown experiment. Figure 4e shows the one-period averaged value of the Poynting vector ($|{\bar{\textbf{S}}}|$) captured at 40 mm downstream from the vortex phase plate, which corresponds to the beam power density and generates the donut-shape beam profile. Additionally, as a preliminary experiment, a beam with a frequency of 28 GHz, power of 210 kW, and pulse width of 1 ms was irradiated only to the vortex phase plate. Similar to a previous experiment^11^, the transmitted beam pattern just after the vortex phase plate was visualized by combining a thermograph sheet with a beam-absorbing sheet, which revealed that the transmitted beam pattern changed to a donut shape. This preliminary experimental results confirmed that the experimentally obtained beam pattern was similar to that obtained via FDTD simulation.

### Optical and impulse measurement setting

The thruster was mounted on a pendulum-type impulse-measurement device to measure the impulse. In this study, the gyrotron device with an internal mode converter was used as the beam source for the thruster. In the gyrotron, the $\hbox {TE}_{42}$ mode induced by a cavity resonator was converted into the quasi-optical mode by the internal mode converter. The waveform and phase were formed using built-in mirrors in the gyrotron, and a Gaussian-like beam was outputted through an alumina output window. This Gaussian-like beam was combined with a corrugated waveguide through a matching optics unit, and the $\hbox {HE}_{11}$ mode propagated inside the corrugated waveguide with an inner diameter of 63.5 mm. Subsequently, the Gaussian-like beam with 28 GHz and 210 kW was emitted in air from the waveguide exit, which was irradiated onto the MITA, as shown in Fig. 4f. Here, the beam power was measured at the open exit of the corrugated waveguide, which included the other modes. However, the purity of the Gaussian component was sufficiently high. The distance between the vehicle and waveguide exit port (*L*) was set to 250 mm. For the parametric study, the impulse measurements were conducted by changing the incident beam pulse width ($\tau$) between 0.6 and 1.5 ms. When the plasma structure inside the cylindrical tube was photographed, the optical window (a blank window) was placed on the tube. An integral image of the plasma was captured using a digital camera (D3300, Nikon) at a resolution of 6000$\times$4000 pixels. However, this optical window was closed when the impulse was measured to confine the shock wave inside the cylindrical tube. The pendulum displacement was measured using a laser-displacement sensor consisting of a sensor head (IL-S100, Keyence), an amplifier unit (IL-1000, Keyence), and a DC power supply (KZ-U3, Keyence). The sensor output signal was obtained by connecting the sensor to a digital oscilloscope (Analog Discovery2, DIGILENT). Based on the pendulum’s equation of motion, the maximum oscillation amplitude can be converted into an impulse as follows:

2 $$\begin{aligned} A_\textrm{max}=\frac{I l_1 l_2}{\sqrt{aM_1-bM_2}gJ}, \end{aligned}$$

where $A_\textrm{max}$ is the maximum oscillation amplitude, *I* is the impulse applied to the vehicle, $l_1$ is the distance between the thrust generation position and fulcrum, $l_2$ is the distance between the displacement measurement position and fulcrum, *a* and *b* are the distances between the center of gravity of lower and upper arms, $M_1$ and $M_2$ are the masses of the lower and upper arms, *g* is the gravitational acceleration, and *J* is the pendulum moment of inertia. Based on Eq. (2), the relationship between the impulse and the measured displacement signal was obtained by applying the known impulse using an impulse hammer. The linear proportionality constant of the calibration line was evaluated as 0.412 mm/mNs, which was used to convert the displacement to impulse data. The impulse measurement was repeated five times and the average value was plotted as the final impulse result. The error bars were evaluated based on the standard deviation of the measured values. The impulse was converted to the momentum coupling coefficient $C_m$, which describes the impulse-generation efficiency of the input beam power. $C_m$ is defined as

3 $$\begin{aligned} C_m=\frac{I}{P \tau }, \end{aligned}$$

where *I* is the impulse, *P* is the beam power, and $\tau$ is the pulse pulse width. By considering the beam divergence between the gyrotron output port and the vehicle’s front, the beam power captured by the vehicle is calculated as

4 $$\begin{aligned} P=0.735 P_0, \end{aligned}$$

where $P_0$ is the beam power at the waveguide exit port. A coefficient of 0.735 was calculated by combining the divergence profile of a Gaussian-like beam with $L=250$ mm, $R_e=20.3$ mm, and a thruster radius of 38 mm. This Gaussian-like beam waist outputted from the corrugated waveguide ($R_e$) was evaluated by combination of theory and experimental measurement, as shown in previous paper^47^. Additionally, the diverging angle of the Gaussian-like beam could be estimated as 0.17 radians. This *P* value was used to evaluate of $C_m$. Here, for direct understanding of $C_m$, $C_m$ can be converted to the impulse with the unit Ns by multiplying the beam energy of $P \tau$. Because $P_0=210$ kW and $\tau =1$ ms are typical values in this study, $C_m=10.0$ N/MW can be converted to the impulse of 1.54 mNs. Although the impulse is intuitive as the force dimension, discussions in this paper were conducted using $C_m$ because $C_m$ is widely used in fields of beaming propulsions.

In this experiment, the working gas was air at 1-atm pressure to understand fundamental behavior. The thruster remained open to atmosphere and air was replaced after pulse irradiation by natural inhalation from the open end downstream of the cylindrical nozzle. Previous simulation^21^ showed the inhalation process from the nozzle’s open end completes within several microseconds; thus, the shot-to-shot interval was set at several minutes, which was sufficient. This experiment required no special device to exchange working gas. However, in future launch missions, beam pulse irradiation and thrust generation will repeat using pulses at 10–1,000-Hz frequency. Here, the inhalation process must terminate quickly. To accelerate inhalation, air intakes will be installed around the vortex phase plate of the vehicle front. When the center body flies at high speed, fresh gas flows through the air intakes and replaces gas around the center body. The next pulse will then be irradiated onto the center body. The experiment with repetitive millimeter-wave beam and optimal thruster design will be discussed in future work.

## Data Availability

Requests for materials or codes should be addressed to Masayuki Takahashi.

## References

[1] Oda, Y. & Komurasaki, K. Plasma generation using high-power millimeter-wave beam and its application for thrust generation. *J. Appl. Phys.* **100**, 113307 (2006).

[2] Oda, Y. et al. Thrust performance of a microwave rocket under repetitive-pulse operation. *J. Propul. Power* **25**, 118–122 (2009).

[3] Oda, Y. et al. A study on the macroscopic self-organized structure of high-power millimeter-wave breakdown plasma. *Plasma Sources Sci. Technol.* **29**, 075010 (2020).

[4] Oda, Y. et al. In-tube shock wave driven by atmospheric millimeter-wave plasma Jpn. *J. Appl. Phys.* **48**, 116001 (2009).

[5] Fukunari, M., Arnault, A., Yamaguchi, T. & Komurasaki, K. Replacement of chemical rocket launchers by beamed energy propulsion. *Appl. Opt.* **53**, 16–22 (2014). 10.1364/AO.53.000I16 25402933

[6] Nakamura, Y. & Komurasaki, K. Theory and modeling of under-critical millimeter-wave discharge in atmospheric air induced by high-energy excited neutral-particles carried via photons. *Plasma Sources Sci. Technol.* **29**, 105017 (2020).

[7] Vikharev, A. L., Gorbachev, A. M., Kim, A. V. & Kolysko, A. L. Formation of the small-scale structure in a microwave discharge in high-pressure gas. *Soviet J. Plasma Phys.* **18**, 554–560 (1992).

[8] Bogatov, N. A. et al. Gasdynamic propagation of a nonequilibrium microwave discharge. *Soviet J. Plasma Phys.* **12**, 415–420 (1986).

[9] Hidaka, Y. et al. Observation of large arrays of plasma filaments in air breakdown. *Phys. Rev. Lett.* **100**, 035003 (2008). 18232990 10.1103/PhysRevLett.100.035003

[10] Tabata, K. et al. Experimental investigation of ionization front propagating in a 28 GHz gyrotron beam: observation of plasma structure and spectroscopic measurement of gas temperature. *J. Appl. Phys.* **127**, 063301 (2020).

[11] Tabata, K., Manabe, A., Komurasaki, K. & Oda, Y. Observation of atmospheric millimeter-wave discharges at 94 GHz and comparison with other microwave and millimeter-wave frequencies. *Appl. Phys. Lett.* **124**, 263903 (2024).

[12] Shimamura, K. et al. Propagation of microwave breakdown in argon induced by a 28 GHz gyrotron beam. *Phys. Plasmas* **28**, 033505 (2021).

[13] Shimamura, K. et al. Wireless power transmission efficiency for microwave rocket using 28 GHz gyrotron. *J. Spacecr. Rockets* **57**, 632–635 (2020).

[14] Takahashi, M. & Ohnishi, N. Computational studies for plasma filamentation by magnetic field in atmospheric microwave discharge. *Appl. Phys. Lett.* **105**, 223504 (2014).

[15] Takahashi, M. & Ohnishi, N. Thrust performance of microwave rocket at low ambient pressure. *Trans. Jpn. Soc. Aeronaut. Space Sci. Aerospace Technol. Jpn.* **14**, Pb_209–Pb_215 (2016).

[16] Takahashi, M. & Ohnishi, N. Plasma filamentation and shock wave enhancement in microwave rockets by combining low-frequency microwaves with external magnetic field. *J. Appl. Phys.* **120**, 063303 (2016).

[17] Takahashi, M. & Ohnishi, N. Numerical study of breakdown pattern induced by an intense microwave under nitrogen and argon gases. *Jpn. J. Appl. Phys.* **55**, 07LD02 (2016).

[18] Takahashi, M., Kageyama, Y. & Ohnishi, N. Joule-heating-supported plasma filamentation and branching during subcritical microwave irradiation. *AIP Adv.* **7**, 055206 (2017).

[19] Takahashi, M. & Ohnishi, N. Open-front approach of a microwave rocket sustained by a resonant magnetic field. *J. Propul. Power* **34**, 762–771 (2018).

[20] Takahashi, M. & Ohnishi, N. Gas propellant dependency of plasma structure and thrust performance of microwave rocket. *J. Appl. Phys.* **125**, 163303 (2019).

[21] Takahashi, M. Coupling simulation on two-dimensional axisymmetric beaming propulsion system. *J. Phys. Conf. Series* **2207**, 012047 (2022).

[22] Takahashi, M. & Ohnishi, N. Thrust-performance maximization of microwave rocket sustained by resonant magnetic field. *Trans. Jpn. Soc. Aeronaut. Space Sci. Aerospace Technol. Jpn.* **17**, 531–537 (2019).

[23] Takahashi, M. & Ohnishi, N. Postural control for beam-riding flight of a microwave rocket using an external magnetic field. *Trans. Jpn. Soc. Aeronaut. Space Sci. Aerospace Technol. Jpn.* **17**, 525–530 (2019).

[24] Takahashi, M. & Ohnishi, N. Gas-species-dependence of microwave plasma propagation under external magnetic field. *J. Appl. Phys.* **124**, 173301 (2018).

[25] Takahashi, M. Development of plasma fluid model for a microwave rocket supported by a magnetic field. *J. Phys. Conf. Series* **905**, 012024 (2017).

[26] Takahashi, M. Asymmetric shock wave generation in a microwave rocket using a magnetic field. *J. Phys. Conf. Series* **905**, 012020 (2017).

[27] Takahashi, M. Microwave-driven in-tube accelerator. *J. Propul. Power*. 10.1038/s41598-025-08430-3 PMC12234760 40624064

[28] Takahashi, M., Yamada, T., Minami, R., Kariya, T. & Shimamura, K. Experimental demonstration of tractor millimeter wave beam propulsion. *Sci. Rep.* **15**, 17544 (2025). 40394191 10.1038/s41598-025-02791-5 PMC12092669

[29] Suzuki, S., Hamasaki, K., Takahashi, M., Kato, C. & Ohnishi, N. Numerical analysis of structural change process in millimeter-wave discharge at subcritical intensity. *Phys. Plasmas* **29**, 093507 (2022).

[30] Suzuki, S., Kato, C., Takahashi, M. & Ohnishi, N. Plasma propagation via radiation transfer in millimeter-wave discharge under subcritical condition. *J. Phys. Conf. Series* **2207**, 012046 (2022).

[31] Suzuki, S. & Takahashi, M. Numerical simulation of electromagnetic-wave interference induced by ionization-front of millimeter-wave discharge at subcritical conditions and application to discharge structure identification. *J. Appl. Phys.* **136**, 153301 (2024).

[32] Takahashi, M. & Ohnishi, N. Beam riding performance of asymmetrically propelled laser vehicle. *AIAA J.* **50**, 2600–2608 (2011).

[33] Takahashi, M. & Ohnishi, N. Beam-riding flight of a laser propulsion vehicle using actively controlled pulse. *J. Propul. Power* **32**, 237–250 (2016).

[34] Takahashi, M. & Ohnishi, N. Theoretical and numerical studies of dynamic scaling of a six-degree-of-freedom laser propulsion vehicle. *Int. J. Aerospace Eng.* **2015**, 801371 (2015).

[35] Takahashi, M., Hayadate, Y., Mori, K. & Hayakawa, A. Free-flight and tracking experiments of a multi-parabola laser propulsion vehicle. *Sci. Rep.* **15**, 15513 (2025). 40319109 10.1038/s41598-025-00429-0 PMC12049452

[36] Sasoh, A. Laser-driven in-tube accelerator. *Rev. Sci. Instrum.* **72**, 1893 (2001).

[37] Takahashi, K. et al. High power millimeter wave experiment of ITER relevant electron cyclorton heating and current drive system. *Rev. Sci. Instrum.* **82**, 063506 (2011). 21721690 10.1063/1.3599418

[38] Beijersbergen, M. W., Coerwinkel, R. P. C., Kristensen, M. & Woerdman, J. P. Helical-wavefront laser beams produced with a spiral phaseplate. *Opt. Comm.* **112**, 321–327 (1994).

[39] Golub, M. A., Shimshi, L., Davidson, N. & Friesem, A. A. Mode-matched phase diffractive optical element for detecting laser modes with spiral phases. *Appl. Opt.* **46**, 7823–7828 (2007). 17994131 10.1364/ao.46.007823

[40] Isakov, D. et al. Evaluation of the Laguerre-Gaussian mode purity produced by three-dimensional-printed microwave spiral phase plates. *R. Soc. Open Sci.* **7**, 200493 (2020). 32874646 10.1098/rsos.200493 PMC7428225

[41] Sawant, A., Choe, M. S., Thumm, M. & Choi, E. M. Orbital angular momentum (OAM) of rotating modes driven by electrons in electron cyclotron masers. *Sci. Rep.* **7**, 3372 (2017). 28611352 10.1038/s41598-017-03533-y PMC5469808

[42] Thumm, M., Sawant, A., Choe, M. S. & Choi, E. M. The gyrotron a natural source of high-power orbital angular momentum millimeter-wave beams. *EPJ Web Conf.* **149**, 04014 (2017).

[43] Thumm, M. Gyro-devices natural sources of high-power high-order angular momentum millimeter-wave. *Trerahertz Sci. Tech.* **13**, 1–21 (2020).

[44] Sawant, A., Yu, D., Kim, D., Choe, M. S. & Choi, E. M. Generation and validation of topological charges of high-power gyrotron orbital angular momentum beams from phase retrieval algorithm. *IEEE Trans. Trerahertz Sci. Tech.* **7**, 164–171 (2017).

[45] Moon, S., Yu, D. & Choi, E. M. High-power millimeter-wave orbital angular momentum mode identification using double slit interference. *IEEE Trans. Plasma Sci.* **52**, 1104–1109 (2024).

[46] Mur, G. Absorbing boundary condition for the finite-difference approximation of the time-domain electromagnetic-field equations, *IEEE Trans. Electromagn. Compat.* **EMC-23**, 377–382 (1981).

[47] Matsukura, M. et al. Instantaneous Measurement of High-power Millimeter-wave Beam for 28 GHz Gyrotron. *Rev. Sci. Instrum.* **90**, 024703 (2019). 30831711 10.1063/1.5050957

